# An engineering perspective on the development and evolution of implantable cardiac monitors in free-living animals

**DOI:** 10.1098/rstb.2020.0217

**Published:** 2021-08-02

**Authors:** Timothy G. Laske, David L. Garshelis, Tinen L. Iles, Paul A. Iaizzo

**Affiliations:** ^1^ Department of Surgery, University of Minnesota, B172 Mayo, MMC 195, 420 Delaware Street SE, Minneapolis, MN 55455, USA; ^2^ Minnesota Department of Natural Resources (retired), 1201 E Hwy 2, Grand Rapids, MN 55744, USA; ^3^ Institute for Engineering in Medicine, University of Minnesota, Minneapolis, MN 55455, USA

**Keywords:** biotelemetry, biologgers, hibernation physiology, wildlife research

## Abstract

The latest technologies associated with implantable physiological monitoring devices can record multiple channels of data (including: heart rates and rhythms, activity, temperature, impedance and posture), and coupled with powerful software applications, have provided novel insights into the physiology of animals in the wild. This perspective details past challenges and lessons learned from the uses and developments of implanted biologgers designed for human clinical application in our research on free-ranging American black bears (*Ursus americanus*). In addition, we reference other research by colleagues and collaborators who have leveraged these devices in their work, including: brown bears (*Ursus arctos*), grey wolves (*Canis lupus*), moose (*Alces alces*), maned wolves (*Chrysocyon brachyurus*) and southern elephant seals (*Mirounga leonina*). We also discuss the potentials for applications of such devices across a range of other species. To date, the devices described have been used in fifteen different wild species, with publications pending in many instances. We have focused our physiological research on the analyses of heart rates and rhythms and thus special attention will be paid to this topic. We then discuss some major expected step changes such as improvements in sensing algorithms, data storage, and the incorporation of next-generation short-range wireless telemetry. The latter provides new avenues for data transfer, and when combined with cloud-based computing, it not only provides means for big data storage but also the ability to readily leverage high-performance computing platforms using artificial intelligence and machine learning algorithms. These advances will dramatically increase both data quantity and quality and will facilitate the development of automated recognition of extreme physiological events or key behaviours of interest in a broad array of environments, thus further aiding wildlife monitoring and management.

This article is part of the theme issue ‘Measuring physiology in free-living animals (Part I)’.

## 1. Background

Biologgers and tracking devices have been used for the past six decades and have provided the principal means of studying the interactions of many free-ranging animals with their environments, including both aquatic and terrestrial species [1–15]. Bears were among the first animals to be radio-collared, in part because their size enabled them to carry large battery packs unencumbered [16]. An interest in bear hibernation also prompted an early use of physiological biologgers, pioneered in the early 1970s using custom-designed devices [17]. Subsequent to this, the field of physiological telemetry emerged, including technological advancements enabling research on both wild and captive animals [6,18–21]. Our team has been using devices designed for human clinical uses, or modifications to such devices, for more than two decades in field investigations focused on heart rate (HR) modulations and the physiology of American black bears (*Ursus americanus*) [22–32]. The principal advantages of using devices designed for humans has been the ability to leverage both advanced features and the exhaustive testing required for the use of such devices in human medicine [33,34]. Yet, for wildlife research applications, challenges can arise since the clinical systems were optimized for characterizing and monitoring human physiology. More specifically, the automatic detection algorithms in the devices were optimized based upon the expected ranges of HRs and rhythms and the frequency characteristics of the human electrocardiogram (ECG). The unique electrophysiologies and anatomies of other species can result in excessive movement artefacts and interfering muscle noise and can cause suboptimal performance of the automatic detection algorithms for rhythm characterization [35]. In addition, the expected ranges for HRs were initially based upon human extremes, which are often exceeded in animals in the wild [4,36–40]. Moreover, the human systems come equipped with a patient activator that allows the given patient-individual to freeze the current ECG in device memory when they experience clinical symptoms. This limits the amount of memory necessary for human clinical applications, but of course, does not translate to wild cohorts.

**Figure 1.**
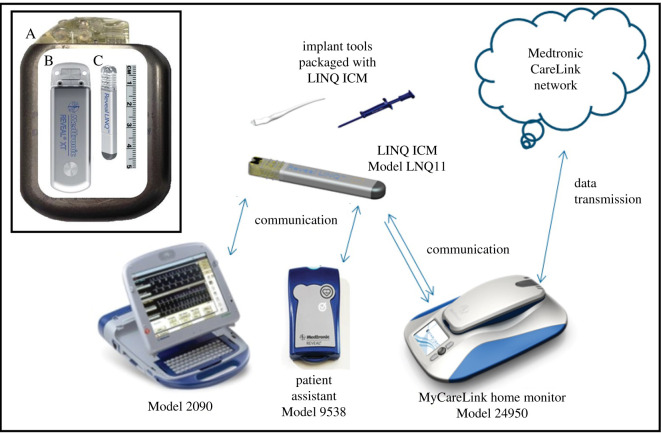
Inset image: The form factors of three generations of implantable monitors used by our research teams. (A) Generation 1 (GEN 1; 80 cc). (B) Generation 2 (Gen 2; 9 cc). (C) Generation 3 (GEN 3; 1.2 cc). The main image (adapted from [41]) shows the systems associated with the human clinical application of the GEN 3 device, including the implantation tools, device programmer (Model 2090), patient assistant, home monitor (Model 24950) and web-based repository (CareLink Network). All system components have been successfully used in wildlife applications, with the exception of the patient assistant, which requires patient interaction to capture episodes of interest.

To date, we have used three unique hardware platforms in our work on bears, Generations 1–3 (GEN 1–3), which were progressively less invasive (miniaturized) and enabled increasingly more comprehensive and consistent data collection (figure 1) [42]. These platforms leveraged both hardware and software developed by Medtronic for their human clinical devices. Where possible, we have shared technical details and specifications, but it should be noted that some product information is considered proprietary by the manufacturer and thus could not be included and/or was not available to our team.

**Figure 2.**
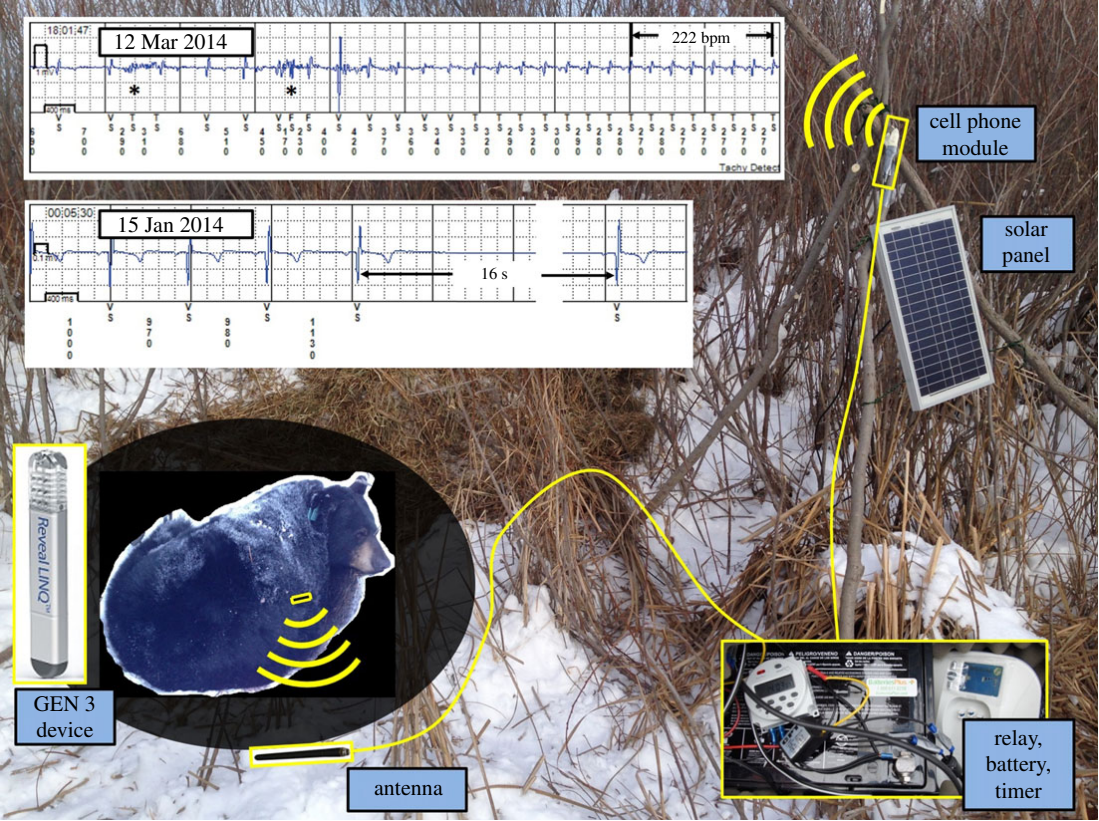
System configuration for a Generation 3 (GEN 3) device with a telemetry station. Antennas with a range of 3–5 m are buried 4–5 cm under the den floor. The antennas transfer the data from the implanted device to the home monitor on a 2 h interval for transmission over a cellular network to a web-based repository. The solar panel-powered system is duty-cycled (turned on for 20 min every 2 h) to enable transmissions while minimizing battery consumption. The sample electrocardiograms (ECGs) shown are from a female black bear in Minnesota hibernating with yearlings. Episodes shown include an episode with a 16 s asystole and a 222 bpm sinus tachycardia that was believed to be related to disturbance of the den by the landowners. The episode with the tachycardia includes two over-sensed beats due to skeletal myopotentials and/or motion artefact (marked with an *).

The monitoring of physiological parameters in combination with location data has provided deeper understanding of how species interact with and adapt to their environment [43]. In our work monitoring HRs and rhythms, respiratory patterns and activities of hibernating bears, we have gained unique insights relative to adaptive physiological mechanisms. We documented extreme variations in HRs, including a 33.8 s asystolic pause and a 261 beats min^−1^ (bpm) sinus tachycardia in our work on black bears [42]. It was also notable that we observed extreme respiratory sinus arrhythmias that act to conserve energy during hibernation, yet provide adequate circulation to maintain the potential for alertness that may be required in the case of a disturbance (i.e. ‘fight or flight’ behaviours) [23]. Long-term data recording has also identified annual trends in HRs and active behaviours associated with denning, parturition, parental care, feeding, migrating and social behaviours [24,27]. Combining physiological data with concurrent GPS collar locations has provided further insights as to the potential impacts of human and environmental stressors (hunting, predation, road crossings, drones), which would not have been apparent through spatial data analyses alone [29–32]. More recently, short-range wireless telemetry has allowed for real-time streaming of data via telemetry stations placed at bears' remote winter den sites (figure 2). Although this perspective focuses on our experiences with American black bears, research leveraging the latter generations of the devices described in this paper has also been carried out in brown bears (*Ursus arctos*), grey wolves (*Canis lupus*), moose (*Alces alces*), maned wolves (*Chrysocyon brachyurus*) and southern elephant seals (*Mirounga leonina*) by our colleagues and collaborators [44–54]. To date, the devices described have been used in fifteen different wild species with publications pending in many instances. In addition to applications relating to conservation and management, opportunities for applying knowledge gained about the unique physiologies of bears to human medicine is an area of increased interest by our team and other researchers. Some of the potential applications to human medicine relating to the physiology of bears, as well as other species, include stroke prevention, elimination of muscle disuse atrophy, treatments for diabetes, heart failure, kidney disease, osteoporosis, respiratory conditions, pre-conditioning organs for transplantation and many others [55–63].

Published papers highlighting results from our biologging studies do not do justice to the bumpy journey of trials and errors that we have experienced. A quote from heart surgeon C. Walton Lillehei and medical device pioneer Earl E. Bakken captures the essence of our trials with bears—“Ready, Fire, Aim: You have to do something before you can find out how to fix the problems that might arise”. Often, we could not foresee potential problems in using medical devices engineered specifically for human applications, so our process was to seek to leverage their capabilities and then adapt when challenges were encountered. Here, we outline the inadvertent misadventures that led to subsequent improvements, and discuss future opportunities that have promise in advancing this area of science.

## 2. Learning from past experiences

In order to provide structure to this portion of the paper and to make it easier for the reader to focus on areas of particular interest, we have used a format that describes the problem encountered and the solutions employed for the various aspects of these systems. Potential future developments and enhancements will be discussed and summarized in §3. The reader should be reminded that the devices were initially optimized for human clinical use and that many of the problems encountered were because our team was pushing the boundaries as to the amount of data to be collected and the duration of data collection, and we were, of course, focused on a species with unique anatomy and physiology. While the solutions that we used were not the only ones available, we present them here to save others from the anguish of lost data and unanticipated obstacles. Here, we recall one of George Santanyana's famous quotes: “Those who cannot remember the past are condemned to repeat it”. In terms of opportunities for improvement, we have focused on modifications to the software programming of the biologgers and other non-implantable components of the system. Since the implantable devices discussed here were designed for human clinical use, further adaptions for our bear research would be costly, and to date, have been beyond the resources available to our team. A summary of the key features of the three generations of implantable devices used by our team is found in table 1.

**Table 1.** Summary of the device generations and associated features.

device	dimensions/mass	bandwidth/sampling rate/sensitivity	recording capacity/battery life	other features
GEN 1	80 cc device volume 20 × 63 × 90 mm 87 g	bandwidth 1–100 Hz 256 Hz sampling rate 8 bits sample^−1^ no autodetection capabilities	104 h electrocardiogram (ECG) recording capacity 96 MB CompactFlashTM memory card 2.45 amp/h	programmable to allow duty-cycling of recording intervals (typically recording EGM for 30 s every 15 min) custom design used componentry from the Jewel Implantable Cardioverter Defibrillator, Medtronic, Inc., Minneapolis, MN, USA. The design is described in detail in the associated publication [22].
GEN 2	9 cc volume 8 × 19 × 62 mm 15 g	bandwidth 0.5–95 Hz 256 Hz sampling rate 16 bits sample^−1^ programmed autodetection parameters: sensitivity 0.035 mV; blank after sense 150 ms	49.5 min ECG recording capacity (using proprietary data compression) 450 MB available for wildlife application 3 year battery life	records daytime HR, night-time HR, activity and episodes with ECGs human clinical device platform: Reveal DX and XT; Medtronic, Inc., Minneapolis, MN, USA [64].
GEN 2 with BearWare	9 cc volume 8 × 19 × 62 mm 15 g	bandwidth 0.5–95 Hz 256 Hz sampling rate 16 bits sample^−1^ programmed autodetection parameters: sensitivity 0.035 mV; blank after sense 150 ms	49.5 min ECG recording capacity (using proprietary data compression) 450 MB available for wildlife application 3 year battery life	GEN 2 features with the following software modifications: Avg HR every 2 min activity at 15 min intervals ECGs for the 10 extremes for tachycardias and asystoles
GEN 3 with telemetry stations	1.2 cc device volume 4.0 × 7.2 × 44.8 mm 2.5 g ± 0.5 g	bandwidth 0.5–95 Hz 256 Hz sampling rate 16 bits sample^−1^ programmed autodetection parameters: sensitivity 0.035 mV; blank after sense 150 ms	57 min ECG recording capacity (using proprietary data compression) 450 MB available for wildlife application 3 year battery life	programmed autodetection parameters: sensitivity 0.035 mV; blank after sense 150 ms records daytime HR, night-time HR, activity and episodes with ECGs wireless transmission on 2 h intervals to telemetry stations placed at winter dens to increase the quantity of ECG data captured during winter hibernation. (Note the product is designed for a single daily transmission and this reduced device longevity from 3 years to approx. 2 years.) human clinical device platform: Reveal LINQTM; Medtronic Inc., Minneapolis, MN, USA [65].
GEN 3 with B-Ware	1.2 cc device volume 4.0 × 7.2 × 44.8 mm 2.5 g ± 0.5 g	bandwidth 0.5–95 Hz 256 Hz sampling rate 16 bits sample^−1^ programmed autodetection parameters: sensitivity 0.035 mV; blank after sense 150 ms	57 min ECG recording capacity (using proprietary data compression) 450 MB available for wildlife application 3 year battery life	GEN 3 and BearWare features with the addition of posture, temperature and impedance at 4 h intervals rejects asystoles > 60 s and tachycardias > 275 bpm

### (a) Data storage and device memory

— Problem: Our original devices required the device to be explanted and a 96 MB SanDisk CompactFlash memory card removed in order to download the data [22]. Solution: Wireless telemetry for data downloading in subsequent generations (devices with up to a 3 m transmission range).
— Problem: Limitations in device memory resulting in overwriting of uniquely recorded data. Solution: Trade-offs were required in what information could be recorded and with what resolution. GEN 1 devices were programmed to cycle to collect windows of data versus continuous recording. In addition, a 21-day delay was programmed to ensure that the bear had returned to a hibernating state prior to the initiation of data collection. In the GEN 2 and GEN 3 devices, we worked with the corporate engineers to determine what information could be collected over a 400-day period before overwriting of memory would occur since this would allow some flexibility around the date of annual den visits/animal handling. We were given discretionary access to a portion of the device memory (approx. 450 MB), which of course required trade-offs in the parameters to be recorded and the recording density. The original GEN 2 and GEN 3 devices could record 49.5 and 57 min of ECGs, respectively, and these devices could also collect single daily values for daytime HR, night-time HR, heart rate variability (HRV) and activity. To better meet our needs, we had the memory reallocated to collect 2 min averages for HRs and activity over 15 min intervals. Also, ECGs were collected for the 10 fastest HRs (this enabled us to document extremes relating to stressors), the 10 most recent HRs exceeding a programmed threshold (this provided insight into the reaction to capture) and the 10 intervals with the longest asystoles (we were interested in how long a bear’s heart can go without beating). GEN 3 had increased functionalities that also enabled the collection of single values for temperature, posture and regional impedance every 4 h. Increasing the recording density for any one of the parameters mentioned would have required a decrease in another parameter. Increasing the frequency of data downloads would of course provide more flexibility in this regard. As mentioned, with our current programming the devices write over existing data when 400 days are reached. We chose this option versus freezing the memory at 400 days in order to always include data that are associated with our most recent interactions with the animals. Another solution was to implant multiple devices, programmed with differing functionalities.
— Problem: The memory cannot be read when the battery reaches the end of life (EOL). Solution: We either replace devices well in advance of EOL or place a second, redundant device, overlapping the battery life of the first device.

### (b) Signal quality and signal processing

— Problem: Poor signal qualities due to suboptimal implant location. Solution: We have found that the left lateral location over the silhouette of the heart is the most reliable in black bears. In our early experiences, we placed a secondary redundant device on the right side of the chest but observed poor signal-to-noise ratios. For other species, we now commonly use smart phone-based applications where an electrode pair can be placed on the skin to seek areas for the best quality cardiac electrograms. In determining the ideal implant location(s), we seek a location and device angulation in which the QRS (the electrical signal corresponding to the depolarization of the right and left ventricles) has an amplitude of at least 0.15 mV and is significantly larger than the amplitudes of both the P- and T-waves.
— Problem: Over- and undersensing of cardiac electrical activities. This is directly related to the fact that these devices were designed and optimized for human clinical applications and both the anatomies and cardiac electrophysiologies vary among species. Solution: The sensitivities of the signal processing algorithms for autodetection of rhythms can be varied to reduce the impacts of muscle noise and/or motion artefacts. We consistently use a sensing threshold of 0.035 mV in our bear research in an attempt to reject such noise. In addition, a ‘blanking period’ can be used to avoid the oversensing of cardiac repolarization (so-called ‘T-wave’ oversensing) where the range of the QT interval (the total duration of the depolarization and repolarization of the right and left ventricles) is known. The blanking period allows the user to set a time interval over which the device will ignore any electrical activity. In bears, we have consistently programmed the devices to a blanking period of 150 ms following the detection of a QRS complex but blanking periods of up to 300 m have been required in other species to avoid T-wave over sensing (TG Laske 2020, unpublished). Having devices that collect and display electrograms versus simply reporting averages, enables the user to troubleshoot sensing issues and confirm the parameters being collected.
— Problem: Oversensing due to myopotentials (electromyographic (EMG) activities from skeletal muscles) or subcutaneous motion artefacts (electrical noise generated by the relative motion of the device and/or the subcutaneous tissues). See an example of this in figure 2. Solution: As is done in human applications, we place the electrodes of the subcutaneous devices towards the skin instead of the muscle and set the sensing threshold at a level that aims to ignore such electrical activity (typically 0.035 mV). In addition, we target implant sites away from large skeletal muscle and regions where we anticipate significant relative motion between the device and the subcutaneous tissues (for example, near intercostal spaces and away from pectoral muscles). We are also careful to avoid teat regions when nursing is anticipated. Although a liability in terms of the sensing of cardiac activity, in several studies we, in turn, have analysed such EMG activations to detect either thermogenic shiver or intense contractions.
— Problem: Lost information during the shipping and transport of explanted devices. When outside of the body, the devices continue to collect both electrical and motion-based data and thus may overwrite relevant information. Solution: Download prior to explantation from the animal or immediately after explantation but before shipping. When this is not possible, ship the devices in a stable package to avoid electrical noise generated by relative motion between the sensing electrodes and packaging.
— Problem: Devices collecting HRs that are outside of a reasonable physiological range due to oversensing, undersensing or transportation. Solution: Programme the devices to ignore implausibly extreme cardiac events. For the American black bear, we programme the devices to reject any asystole (a period of no heart activity) of longer than 60 s and any tachycardia (a period of a high HR) exceeding 275 bpm. In an early iteration of our wildlife-modified software, we programmed the devices to stop collecting data if a heartbeat was not detected for 2 min (surmising that the animal had died). Unfortunately, we found that in situations of extreme physical activity, the subcutaneous electrical signal can become weak and undetected for several minutes and thus we had devices turn off in healthy, active animals.
— Problem: Inadequate specificities on high HR episodes. Solution: A key programming feature for high rate episodes is to require a minimum number of beats to exceed a particular rate for the episode to be considered a true tachycardia. We currently require 48 consecutive beats above the programmed threshold for the event to be recorded. This helps to avoid the recording of transient noise due to myopotentials or motion artefact.
— Problem: Improper rhythm characterization. Solution: When programming the devices, an awareness of both the anticipated extremes and rhythm characteristics are key to selecting the appropriate programmed parameters. As an example, we turn off atrial fibrillation (AF) detection within the human clinical devices. The associated human algorithm looks for unusual variations in the ventricular HR, and due to the extreme respiratory sinus arrhythmia that commonly occurs in bears but is very mild in humans, the device believes that the bear is in AF nearly all of the time. Another example of a species-specific extreme relates to asystoles. The human clinical devices can be programmed to set the asystolic sensing threshold to a maximum of 4.5 s. It is noteworthy that, in a hibernating bear, the asystole exceeds 4.5 s with nearly every respiratory cycle. To compensate for this, we now programme the devices to catalogue the 10 longest periods without a heartbeat that are longer than 4.5 s and shorter than 60 s. This gives us a snapshot of the extremes. In a similar manner, we also have the devices collect the 10 fastest HR episodes of more than 176 bpm and less than 275 bpm.
— Problem: During hibernation, HRV was often recorded as ‘zero’. Solution: The devices utilized report HRV as the standard deviation of the median HRs over a 5 min interval. The so-called SDANN method provides insight into daily variations in HRs, but this particular algorithm is not designed to detect and record beat-to-beat variations [66]. In the bears, although the beat-to-beat variation in HRs during hibernation is extreme, the overall daily median HRs are extremely consistent and hence HRV is reported as zero. Clearly, an intimate knowledge of both the physiological parameters of interest and the signal processing methodologies used by a given device is critical to both data interpretation and the collection of the most relevant information.
— Problem: Lack of temporal resolution on recorded data limited the ability to correlate GPS locations and activities with specific physiological responses. GEN 1 was limited to 104 h of wide-band electrograms, and GEN 2 and 3 clinical software only provided average daytime and night-time HRs and daily activity. Since the human clinical devices were designed to focus on specific arrhythmogenic events and general trends in HRs and activities, reallocation of memory and new data sampling algorithms were required for wildlife application. Solution: The team developed custom software for use with the GEN 2 and GEN 3 devices to record 2 min averages for HRs and activity for 15 min intervals. In addition, non-commercially available algorithms and sensors were used to record subcutaneous temperature, impedance and posture on 4 h intervals. See table 1 for additional detail.
— Problem: Clock in the device does not match other devices or systems (e.g. time recorded for GPS locations). Solution: Check and record the exact time in the biologger at the time of implantation and record the exact device time during each data download since some drift may occur. As an example of where temporal synchronization becomes problematic, the devices used do not adjust for daylight savings time and this may create confusion when comparing to other data sources (such as behavioural observations).

### (c) Data transmission and downloading

— Problem: Poor communication with the implanted device when attempting a download. Solution: When two devices are implanted in an animal, the auto-device detection used by the telemetry head can become confused. This is remedied by using a metallic object (clipboard, surgical tray, etc.) to isolate the targeted device from a secondary device. When monitoring remotely with telemetry stations, only a single device is used and we use two antennas split onto separate coaxial cables inside the den. This both improves the likelihood that the animal will be close enough to one of the antennas to communicate, but also provides redundancy if one is damaged (e.g. chewed by bears).
— Problem: Variable or poor cellular reception for wireless data transmissions at bear dens. Solution: Check whether the den site has adequate cellular coverage, and from which provider, via network maps. Ideally, you should also visit the site prior to hauling in the equipment since areas purported to have adequate cellular coverage may not when you are in a deep ravine or similar.

### (d) Power in the field

— Problem: The instrumentation used for communicating with the implanted devices was designed for use in medical clinics and most commonly is line powered. Solution: We operate the equipment on 12 V batteries with a DC to AC power inverter. We carry 14–21 amp h 12 V sealed lead-acid batteries in backpacks for short term use. For long-term deployment, we use two 55 amp h 12 V sealed lead-acid batteries connected in parallel, which are transported over the snow on a sled or carried in backpacks.
— Problem: Telemetry station battery drain. Solution: We connect solar panels to the batteries that are powering remote telemetry stations and use a controller to avoid overcharging and battery drain back to the panels. In this application, we also duty cycle the equipment such that it is only powering the equipment when required (we transmit on a 2 h interval and thus only power on the equipment for a 20 min period during this interval).

### (e) Implant stability and retrieval

— Problem: Devices rejected due to a foreign body response. Although we have tried numerous implant techniques, we have found that the American black bears often elicited an extreme foreign body response and commonly rejected devices through the skin (even devices that had remained successfully implanted for up to 2 years). This phenomenon has been reported in more detail by our team and by previous investigators [25,67]. Moreover, we found that rejection was particularly high for devices implanted in summer, or early denning. We have sometimes found devices at den sites with a metal detector. Interestingly, this is not common in brown bears [42]. Solution: Implant devices during late denning. Implant two devices for redundancy (we place both devices on the left side of the bear to optimize signal quality). Use smaller ‘injectable’ devices (GEN 3), which have been found to reduce rejection rates in black bears in our experience. As a comparison, in human clinical use, erosion/rejection of the implant site was reported in 1.2% of patients in a study using the GEN 2 device [68]. For the GEN 3, rejection of this device was reported in 0.4–0.6% of clinical patients, with one device noted to have externalized due to trauma, which may be a common factor contributing to device rejection in the wild (*N* = 1/451) [69,70].
— Problem: Retrieval of devices from animals legally shot by hunters or killed in motor vehicle accidents. Solution: Animals have ear tags with contact information, so we often had an opportunity to talk to hunters or drivers about retrieving devices.

## 3. Future opportunities

For more than two decades, our research team has benefited from the use of modern implantable physiological monitors designed initially for human clinical applications. In addition, in a partnership with corporate biomedical engineers and scientists, we have developed custom software applications for these devices to expand their utility. Although this has resulted in significant advancements in our ability to monitor free-ranging black bears, exciting opportunities remain both to address current limitations and to expand functionalities across wider taxa and environments.

As we look forward to the future, numerous issues can be readily solved. Importantly, new opportunities will be presented by the expanded use of wireless telemetry and cloud-based data storage. Such data transfers can occur via wearable transponders or via telemetry stations/transponders placed in a location where animal presence is expected [71]. Importantly, combining these technologies will dramatically reduce the on-board data storage requirements and potentially further decrease device sizes, enabling use on ever-smaller species. It should be noted that the effects of biologgers on small, mobile species (e.g. birds) remain a concern and researchers must weigh such effects against both the welfare of the animals and the impact on the hypotheses under investigation [72,73]. In addition, if the entire content of the device memory can be transmitted, manual downloads for retrieving the full complement of data, and the related handling of the animals, would be minimized or even no longer be required. Reducing handling of both wild and captive species, of course, reduces the impact on behaviours and physiology and also reduces risks associated of both handling and anaesthesia [74,75]. Further, it would be ideal if the implantable devices could be reprogrammed remotely. This would allow real-time adjustments to programmed parameters in the event of inappropriate sensing and/or changes in the types of data to be collected. This would be particularly important in species where pilot work/benchmarking on captive populations is not feasible (for example: cetaceans) and/or where physiologic extremes were not anticipated/documented prior to device deployment (for example: species prone to predation and/or experiencing ‘fight or flight’ situations).

As previously described, wireless telemetry and cloud-based storage were used in the GEN 3 devices for partial downloads of device memory (figure 2). Stationary monitors that were either line powered or powered within the aforementioned telemetry station were required [26,28]. In these instances, either the monitor or an employed antenna extension must be within 3–5 m of the implanted subject. We have found that such stationary monitors have had significant practical uses for studying hibernating American black bears that remain at their den site all winter and typically do not abandon dens when monitoring devices are installed. These same monitors have also been applied to captive species where the location of the animal is predictable [53]. Further, such monitors could also be deployed to download data when the return of an animal to a particular location is predictable; such as is the case with many species of nesting birds.

**Figure 3.**
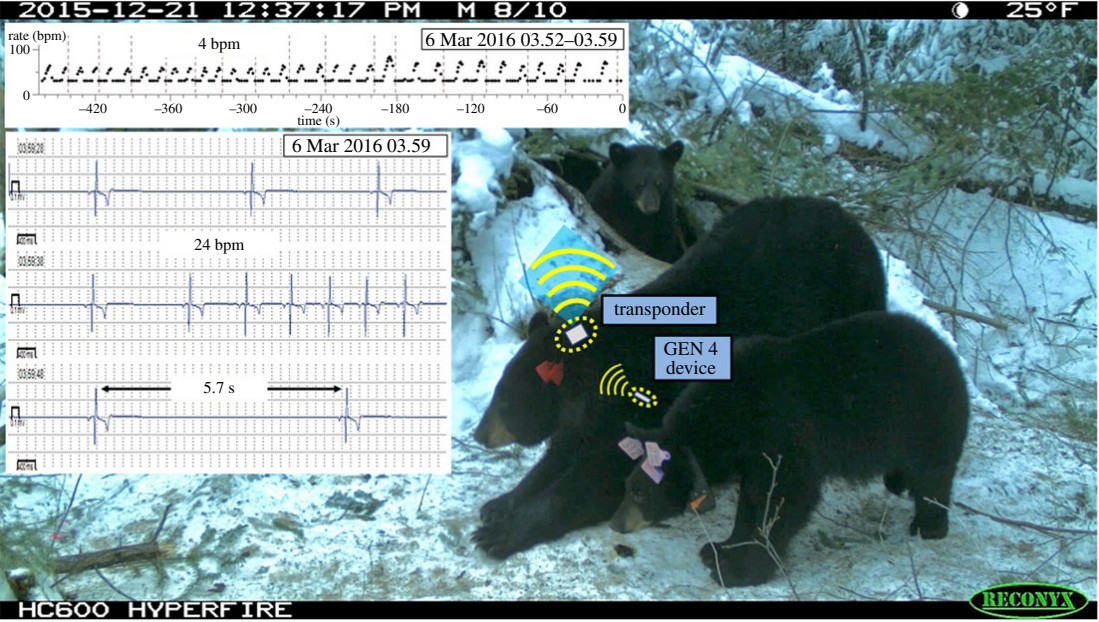
Proposed configuration for the GEN 4 system. The implanted device transmits data to a radio collar-mounted transponder, which in turn communicates with either a cellular or Iridium network. This allows data transfer from an ambulatory/free-ranging animal to a web-based repository. The associated photo was captured by a trail camera at the den site during an active period where the mother and two yearlings had emerged from the den. The upper graph plots the heart rate over an 8 min interval occurring while this female was in the den hibernating. The variations in heart rate are due to a respiratory sinus arrhythmia, and thus the respiration rate can also be calculated (one heart rate cycle per respiration). The 30 s electrogram shown is for the same time period, allowing a detailed analysis of the rhythm. Although these exemplary data were captured by a stationary GEN 3 telemetry system, similar data capture and transfer are anticipated with GEN 4.

The device to transponder link in our next-generation system (GEN 4) uses the Bluetooth protocol, enabling data transfer and communication across a distance of 3–5 m to smart devices (cell phone, tablets, etc.) instead of the current stationary wall-powered monitor. Ideally, these transponders will have a life similar to the implanted monitor and can be mounted onto radio collars or worn by the animals for ambulatory monitoring and geolocation information [76]. For species where a mounted transponder is not practical, telemetry stations might again be applied in locations where, at the least, the transient presence of the animal is predictable, or even attached to a drone to enable an approach for data transfer. Upon transmission of data, memory can then be reallocated and reused, thereby enabling dramatic increases in the information collected. However, an increase in the on-board data storage would enable the collection of significantly more data between transmissions. An example of a situation requiring increased memory would be monitoring continuous ECGs and/or detailed data on other physiological parameters; these could be stored in short term memory on either the device or transponder and then transmitted routinely. It is anticipated that data could then be streamed either on programmed intervals or ‘on-demand’. This has broad application across species and would eliminate current compromises required due to either intermittent/duty-cycled data collection or data compression. In other words, this could eliminate sampling biases due to seasonal data collections and/or the non-detection of rapid physiological modulations in behaviours. Finally, a means of synchronization of all device clocks (or regular clock updates during transmissions) should be considered for any automatic modality for data transmission to ensure temporal alignment with other systems, such as GPS collars. Figure 3 shows the future configuration that will be enabled by the GEN 4 device.

Considerations for transmission to a cloud-based repository will require either local cell phone coverage or satellite transmission via the Iridium, Globalstar or Starlink satellite networks, or in some geographies, via the 5G network. Although cell phone coverage has become increasingly ubiquitous, there are still many regions on the planet with no coverage or where poor coverage exists for one or all providers. This problem was solved for animal tracking collars by using satellite networks for data downloads, thereby reducing the chances of lost data (animals with store-on-board data not recaptured) and expanding the usefulness of data collected in real-time [77]. Nevertheless, diving mammals represent a unique challenge, where the link to even a satellite network is transient. It is our hope that the transition to 5G will enable fast, data-intense uploads during short transmission windows.

As wireless transmissions become more frequent, biologger power consumption will also increase and so concurrent improvements will be required in battery capacities or means of recharging might be deployed. Small solar panels or circuits that transform mechanical energy from motion into electrical energy (piezoelectric or mechanical technologies, such as is used in watches) could be incorporated into such transponder systems [78–80]. In the event of battery failure or exhaustion, permanent, retrievable memory would be desirable. In some species, reductions in size may be the most valuable improvement feature, but in bears and other larger mammals, increases in battery size/capacities and memory and/or an increase in inter-electrode spacing would likely provide a better trade-off regarding longevities, functionalities and specificities of sensing. Note that recommendations on the maximum mass of biologgers vary by taxa [81]; for example, they should not exceed 3–5% of the mass of flying species [72,82–84], but even below this threshold negative effects have been observed in some species during sensitive periods [73,75]. This would make the ongoing GEN 3 device applicable to individuals as small as 83 g. With decreases in device size, researchers have continued to push the envelope to enable monitoring of ever-smaller species [85]. For many species, where externally mounted devices could interfere with movement, implantable devices may be beneficial [73,86]. In any circumstance, a thorough understanding of the impact of the biologger on the viability of the animal and the particular behavioural aspects under study is always recommended [82,87–92].

Significant unmet needs in relation to the use of human clinical devices and systems in the field include weather-, environment- and animal-proofing. Current human clinical systems are designed to be used in controlled environments, and thus we have had programming systems and monitors (installed at bear den sites) fail due to cold and wet conditions. To minimize the burden associated with transporting programmers in the field, tablet-based programmers are emerging, but these are typically also designed for in-home use and normally will not function well in the cold. As is the case for any systems used in the field, the user needs to protect the integrity of the system from both the environmental extremes and from the animals themselves (e.g. in our bear research we found that the animals were inclined to chew or tamper with the systems left at their den site). The use of such systems in diving mammals must also consider the impact of extreme pressures on the integrity of both implantable and wearable devices.

One of the chief restrictions that we encountered with human medical devices is that they are specifically targeted to the characteristics of human HRs, anatomies and associated electrophysiology. We have adapted the programming to accommodate the wide range of HRs seen in bears, but more flexibility in this regard would be desirable, especially as use expands to other species. Some examples of natural extremes that have been reported in the literature include blue whales with average HRs of 25–37, and 4–8 bpm during a dive, and hummingbirds and bats with HRs in the range of 500 bpm at rest to more than 1000 bpm in flight [36,39,40]. Such rates are not within the range of human applications and thus the data processing or data displays do not readily accommodate these extremes (for example, GEN 2 and 3 only show trends for average HRs of 30 bpm or greater). Although the devices we used have typically been found to properly interpret the electrical signals in bears, the algorithms were found to be unreliable in a recent study on moose [52]. When adapting any device to a new species, it is of course helpful to conduct pilot trials in captivity to both understand the benefits and limitations of a particular technology. Pilot evaluations in captive cohorts not only allow for optimization of telemetry and biologger system designs, but also for the determination of the best procedural techniques and implant locations. This approach is being taken in captive cohorts of black bears, grey wolves, maned wolves and scimitar-horned oryxes (*Oryx dammah*) [51,53].

Transmission/streaming of raw signals would allow unlimited possibilities, as well as creating repositories to feed machine learning algorithms. Such transmissions would reduce the challenges of interspecific differences in biology or cardiac electrophysiology, since they could be managed through post-processing. A practical example of this would relate to transmissions of continuous wide-band ECGs. Post-processing could ensure that no over- or undersensing of HRs occurs due to myopotentials/poor signal quality and/or species-specific cardiac electrophysiology [93]. From these recordings we can also measure respiratory rates via cyclic modulations of the QRS signal amplitudes as the lungs inflate/deflate (thoracic cavity diameter increases), thus enabling the potential for calculations of metabolic rates [24,94]. Instead of rejecting myopotentials, one can use them as another surrogate for activities. In GEN 1, we used post-processing on such electrograms to calculate basic electrophysiological parameters, including PR (the interval from the initiation of the depolarization of the right and left atria to the initiation of the depolarization of the right and left ventricles) and QT intervals and QRS durations. These calculations could be achieved by novel algorithms within the next-gen device, but an advantage of cloud-based computing is that it allows not only prospective analyses, but retrospective analyses of existing data using novel methods. One such specific example relates to heart rate variability (HRV), where the devices currently calculate HRV using the SDANN method, storing a single daily value. Access to raw data would allow HRV analyses using any method on a beat-to-beat basis.

As biologger technologies evolve, the potential exists for more forms of real-time monitoring. These might be in the form of on-demand data access to query the current physiological state as well as the given location of the study animal. It might also be in the form of an ‘alarm’ when certain programmed physiological parameters are exceeded, much like the currently available alarms on GPS tracking collars that signal, in real-time, events such as deaths, birthing, migrating or movements into or outside a certain defined area [77]. The animal's given physiological state combined with locational data could provide researchers with real-time indications of stressful conditions, enabling immediate on-site field investigations.

Collectively, the aforementioned advances can and will continue to provide quantum leaps in our understanding of the physiologies and behaviours of numerous species of free-ranging animals. Combined with the rapidly advancing animal tracking devices, they will be part of what Kays *et al*. [77] have called the ‘golden age … of unprecedented exciting discoveries'. Increasingly efficient biologgers and advanced data transfer protocols will provide an onslaught of detailed information, requiring increasingly sophisticated analytical tools to keep up and decipher all that the data can divulge. We look forward to not only working to advance scientific and conservation efforts through future advancements in biologger technologies, but also to solving the misadventures that are sure to accompany the evolution of such devices.

## Data accessibility

This article has no additional data.
